# Nod2 is required for the early innate immune clearance of *Acinetobacter baumannii* from the lungs

**DOI:** 10.1038/s41598-017-17653-y

**Published:** 2017-12-12

**Authors:** Sandeep D. Kale, Neha Dikshit, Pankaj Kumar, Vanniarajan Balamuralidhar, Hanif Javanmard Khameneh, Najib Bin Abdul Malik, Tse Hsien Koh, Gladys Gek Yen Tan, Thuan Tong Tan, Alessandra Mortellaro, Bindu Sukumaran

**Affiliations:** 1 Program in Emerging Infectious Diseases, Duke-NUS Medical School, Singapore, 169857 Singapore; 2 Singapore Immunology Network (SIgN), Agency for Science, Technology and Research (A*STAR), Singapore, 138648 Singapore; 3 Department of Microbiology, Division of Pathology, Singapore General Hospital, Singapore, Singapore; 4 DSO National Laboratories, Singapore, Singapore; 5 Department of Infectious Diseases, Singapore General Hospital, Singapore, Singapore

## Abstract

*Acinetobacter baumannii* (*A*. *baumannii*) is a significant cause of severe nosocomial pneumonia in immunocompromised individuals world-wide. With limited treatment options available, a better understanding of host immnity to *A*. *baumannii* infection is critical to devise alternative control strategies. Our previous study has identified that intracellular Nod1/Nod2 signaling pathway is required for the immune control of *A*. *baumannii* in airway epithelial cells *in vitro*. In the current study, using Nod2^−/−^ mice and an *in vivo* sublethal model of pulmonary infection, we show that Nod2 contributes to the early lung defense against *A*. *baumannii* infection through reactive oxygen species (ROS)/reactive nitrogen species (RNS) production as Nod2^−/−^ mice showed significantly reduced production of ROS/RNS in the lungs following *A*. *baumannii* infection. Consistent with the higher bacterial load, *A*. *baumannii*-induced neutrophil recruitment, cytokine/chemokine response and lung pathology was also exacerbated in Nod2^−/−^ mice at early time points post-infection. Finally, we show that administration of Nod2 ligand muramyl dipeptide (MDP) prior to infection protected the wild- type mice from *A*. *baumannii* pulmonary challenge. Collectively, Nod2 is an important player in the early lung immunity against *A*. *baumannii* and modulating Nod2 pathway could be considered as a viable therapeutic strategy to control *A*. *baumannii* pulmonary infection.

## Introduction

The aerobic gram-negative bacillus, *Acinetobacter baumannii*, has emerged as a major global health threat^1,2^. Pneumonia, blood-stream infections, urinary tract infections, meningitis and burn- wound infections are some of the clinical manifestations associated with *A*. *baumannii* infection^3^. Although generally associated with nosocomial infections, *A*. *baumannii* can cause severe community-acquired infections as well^1,4^. Hospital-acquired pneumonia is the most severe clinical manifestation associated with *A*. *baumannii* ^3,5^. The rapid rise in the multi-drug resistant and pan-drug resistant forms of *A*. *baumannii* is truly alarming^2^. *A*. *baumannii* topped the World Health Organization’s recent list of bacterial pathogens for which new treatment options are critically needed (http://www.who.int/mediacentre/news/releases/2017/bacteria-antibiotics-needed/en/). Despite its clinical importance, there has been only very slow progress in the development of control strategies against this emerging threat. With few anti-microbials in the developmental pipeline, alternative or adjunct strategies are desperately needed. *A*. *baumannii* generally poses no threat to healthy individuals, but causes serious infections in immunocompromised and intensive care populations, suggesting a major role for the innate host defense in the infection control. Understanding the interaction of *A*. *baumannii* with the host immune system is very important in the development of newer strategies (such as immunotherapy) to control these infections.

Activation of the innate immune response following *A*. *baumannii* lung infection leads to the secretion of proinflammatory cytokine and chemokine secretion and subsequent recruitment of immune cells to the lungs. Neutrophils constitute the major and the critical immune cells that are recruited to the lungs^6,7^ during *A*. *baumannii* pneumonia followed by macrophages^8^ and NK cells^9^. The microbicidal strategies known to be involved in early *A*. *baumannii* killing includes reactive oxygen species (ROS), myeloperoxidase and β-defensin-2 with reactive nitrogen species (RNS) playing only a minor role^10–12^. Qiu *et al*.^12^ demonstrated that the early killing of *A*. *baumannii* by the resident phagocytes and inflammatory cells depends on the presence of an intact phagocyte NADPH oxidase, thus suggesting a crucial role for the oxidative burst in the rapid killing of ingested *A*. *baumannii* by phagocytes. However, the innate immune signaling pathways involved in ROS production during *A*. *baumannii* pneumonia are not known.

Although a few studies have reported the involvement of innate immune pathways in controlling *A*. *baumannii* pulmonary infections, a comprehensive understanding of the relative contribution of these pathways is still lacking. Being a Gram-negative bacterium, LPS and the TLR4 pathway contributes to the cytokine/chemokine secretion and neutrophil recruitment during *A*. *baumannii* lung infection^10^. TLR2 has been shown to play either a protective or detrimental role during *A*. *baumannii* lung infection^10,13^.

Although generally viewed as an extracellular bacterium, *A*. *baumannii* is capable of invading mammalian cells^14–16^, highlighting the important role for the intracellular innate immune recognition in the immune defense against *A*. *baumannii*. However identification and characterization of the role of intracellular innate immune receptors in *A*. *baumannii* infections is still in its infancy. Using pneumonia and systemic murine infection models, a recent report has revealed an important protective role for the intracellular innate immune sensor TLR9 in *A*. *baumannii* immune response^17^. Our recent study showed that the NLRP3 inflammasome plays an important role in the pulmonary host defense against *A*. *baumannii* clinical isolate, but not against *A*. *baumannii*-type strain^18^. Using lung epithelial cells and *in vitro* infection model systems, our group has also shown that the intracellular innate immune receptors Nod2, Nod1 and Rip2 are important innate immune receptors that recognize intracellular *A*. *baumannii* ^14^. In the current study, we sought to determine the *in vivo* relevance of Nod2 in the innate immune and the inflammatory response associated with *A*. *baumannii* using a sub-lethal pneumonia infection model in mice. Our study demonstrated that Nod2 is critical for the early but not the late innate immune control of *A*. *baumannii* pneumonia. Mechanistically, Nod2 contributed to the oxidative stress generation (ROS/RNS) for *A*. *baumannii* control during early time points of infection.

## Results

### Nod2-deficient mice are more susceptible to *A*. *baumannii* at early time points of infection

**Figure 1:**
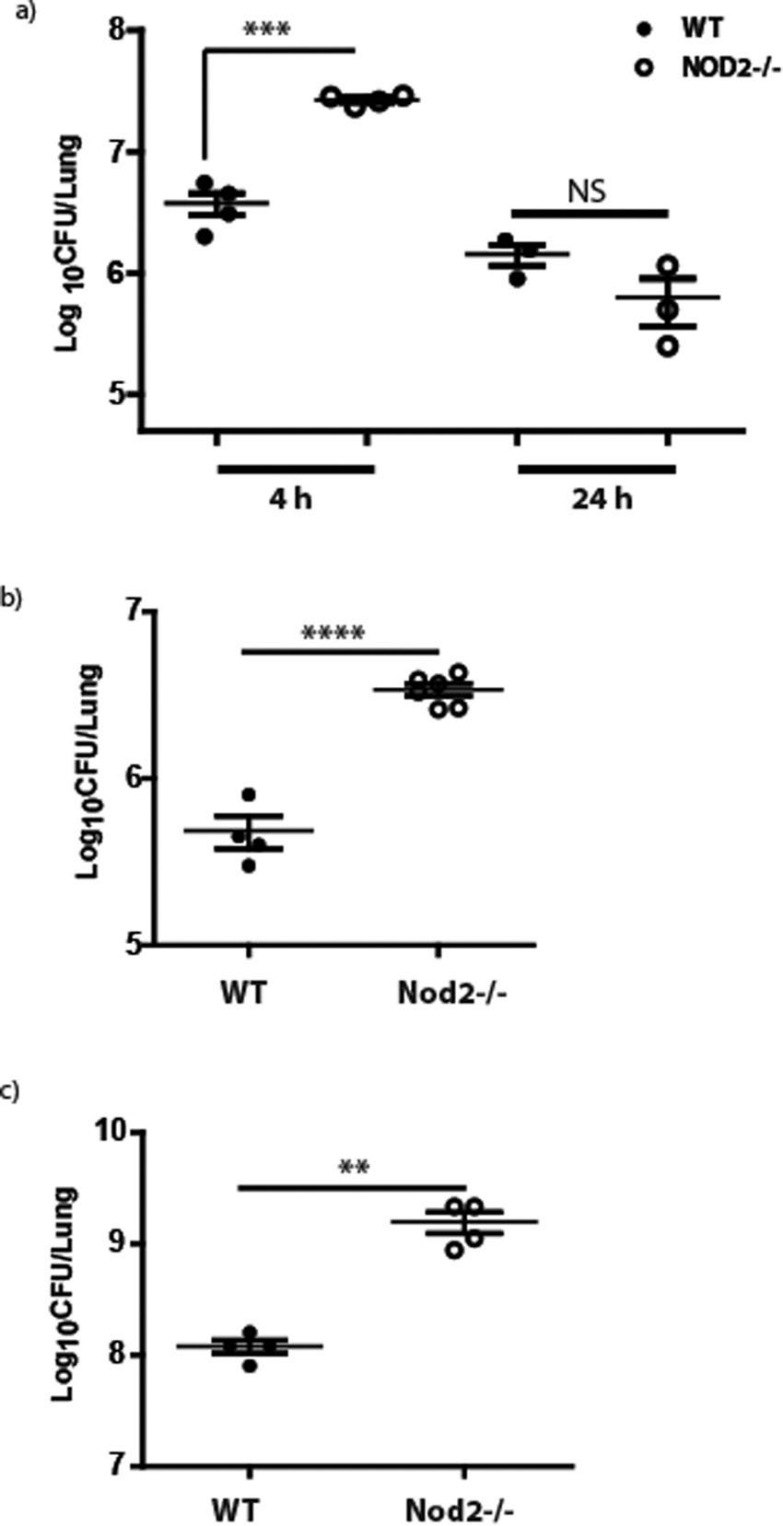
Nod2 is involved in early clearance of *A*. *baumannii* from lungs. Wild type (WT) or Nod2^−/−^ mice were infected with *A*. *baumannii* (n = 4–6 mice per group). At designated time points bacterial cfu was enumerated in lungs as described in material and methods and the bacterial load in lungs was compared between the 2 groups at 4 (**a** and **c**), 12 (**b**), and 24 h (**a**) post infection. The confirmed bacterial inoculation doses were 1 × 10^7^ (a and b) and 2 × 10^7^ (**c**) respectively. **Represents *p* ≤ 0.01, ***represents *p* ≤ 0.001, ****represents *p* ≤ 0.0001. Values shown represent mean ± standard deviation of results of three independent experiments.

To determine the *in vivo* role of NOD2 in the pulmonary host defense against *A*. *baumannii*, we used a sub-lethal murine pulmonary infection model using the ATCC strain 19606 (AB-19606). Based on previous published reports from other research groups, and our data^18^, a sub-lethal (5 × 10^7^ cfu) mouse pneumonia model for *A*. *baumannii* infection in C57BL/6 mice was used for the studies. Nod2^−/−^ mice or control wild type C56BL/6 mice were intranasally infected with *A*. *baumannii* and the bacterial load was determined in the lungs at early (4 h) and late (24 h) time points post-infection. As seen in Fig. 1a, the pulmonary bacterial load was significantly higher in Nod2^−/−^ mice compared with wild type controls during the early time point (4 h) post-infection (*p* = 0.0002). However, at 24 h post-infection, the bacterial loads were comparable in Nod2^−/−^ and wild type mice. AB-19606 was cleared from the lungs of all the infected mice ~48 h post-infection. To further confirm the role of Nod2 in the early innate immune control of *A*. *baumannii* in the lungs, we also compared the *A*. *baumannii* load between the wild type and Nod2^−/−^ mice at an additional early time point post-infection (12 h). The result shown in Fig. 1b show that the pulmonary bacterial load is significantly higher in Nod2^−/−^ mice in comparison to wild type controls at 12 h post-infection. From this data, we conclude that Nod2 may play important role in the *A*. *baumannii* control at the early stages of pulmonary infection, but not at the late stages.

We further assessed the role of Nod2 pathway in the host resistance to more virulent *A*. *baumannii* strains. For this, we selected AB-8879 (an *A*. *baumannii* clinical isolate belonging to international clonal group II)^18^. The results given in Fig. 1c shows that Nod2 deficient mice has higher lung bacterial load in comparison to wild type controls. This result further validates that Nod2 pathway may also important in the host resistance to more virulent strains of *A*. *baumannii*.

### *A*. *baumannii*-induced early recruitment of neutrophils to the lungs is enhanced in Nod2−/− mice

**Figure 2:**
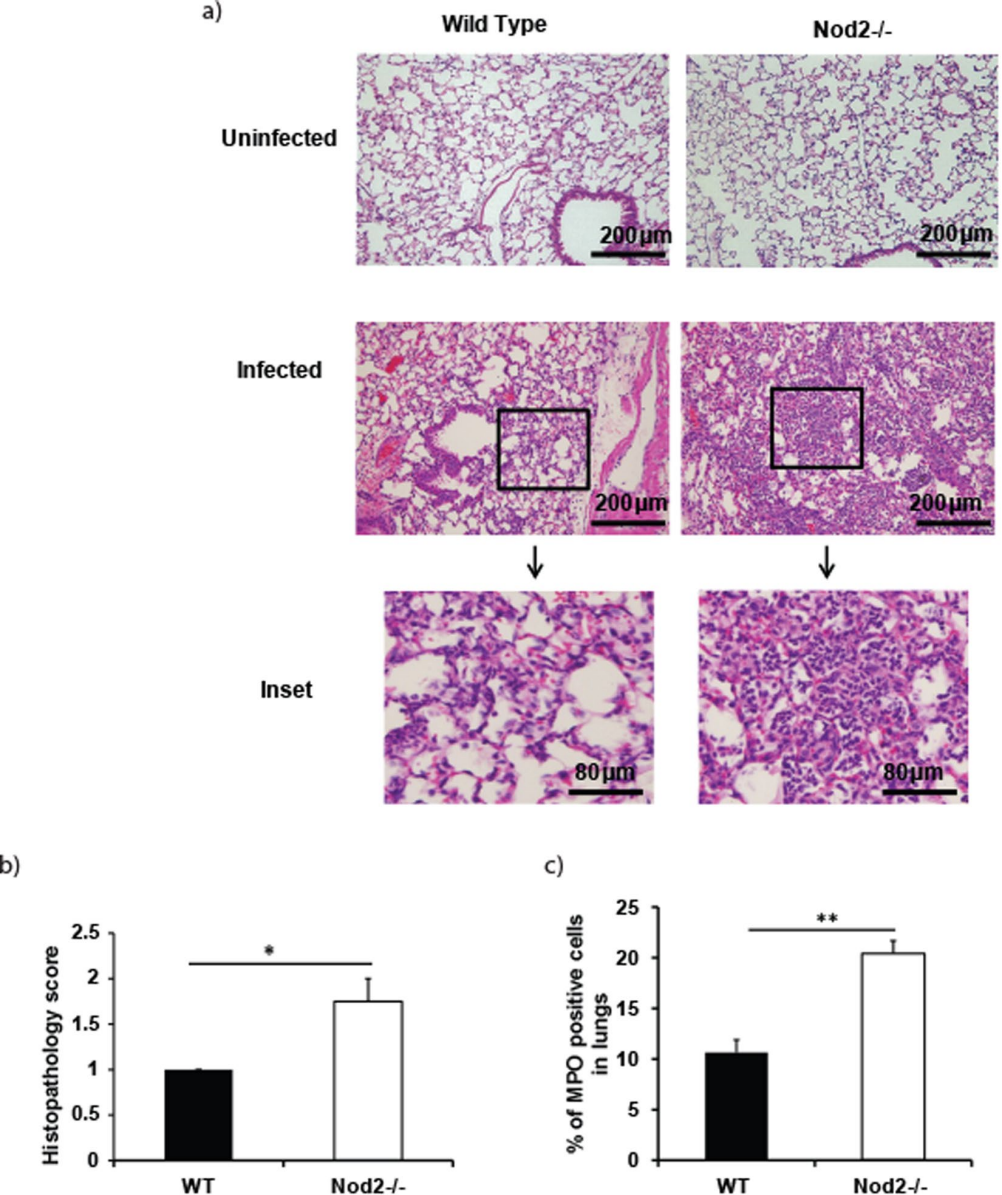
Lungs of *A*. *baumannii* infected Nod2^−/−^ mice show higher pathology and neutrophil influx as compared to lungs of infected WT mice. WT or Nod2^−/−^ mice were intranasally instilled with *A*. *baumannii* or PBS control (uninfected). At 4 h post infection mice were euthanized. (**a**,**b**) Paraffin embedded lung sections were stained with haematoxylin and eosin and were evaluated for pathology and neutrophil infiltration as described in material and methods. (**a**) Lungs of infected Nod2^−/−^ mice had higher neutrophil influx as compared to lungs of infected wild type mice. Shown are representative images (10X magnification, scale bar represents 200 µm). Inset shows higher magnification image (scale bar represents 80 µm). (**b**) Lungs of infected Nod2^−/−^ mice showed higher pathology as compared to lungs of infected wild type mice. (**c**) Paraffin embedded lung sections were stained for MPO as described in material and methods. Lungs of infected Nod2^−/−^ mice had higher MPO positive cells as compared to lungs of infected wild type mice. *Represents *p* ≤ 0.05. **Represents *p* ≤ 0.01, Values (**b** and **c**) shown represent mean ± standard deviation (n = 4–5 mice/group) and is representative of three independent experiments.

Early recruitment of neutrophils, the key immune cells, to the lungs is essential for the clearance of pulmonary bacterial pathogens including *A*. *baumannii*. In addition, Nod2 deficiency has been previously shown to delay neutrophil recruitment to the lungs following bacterial exposure^19,20^. To determine if Nod2 plays any role in the early recruitment of immune cells to the lungs during *A*. *baumannii* infection, we assessed lung immune cell recruitment in Nod2^−/−^ mice in comparison to wild type controls. Histopathological analysis of the mouse lungs prior to infection did not reveal any significant difference between wild type and Nod2^−/−^ mice (Fig. 2a). However, significant number of neutrophil infiltration (perivascular, peribronchiolar and/or alveolar) in the lungs was observed in all the mice during *A*. *baumannii* infection at early time points (4 h). Moreover, at 4 h post infection, the severity of neutrophil infiltration in Nod2^−/−^ was significantly higher in comparison to wild type mice {Fig. 2b shows histopathological scoring) and Fig. 2a shows the representative H&E stained section}. Using MPO assay, we further confirmed the initial enhanced neutrophil recruitment observed in the lungs of Nod2^−/−^ mice during *A*. *baumannii* infection (Fig. 2c). Thus the enhanced neutrophil infiltration into the lungs of *A*. *baumannii* infected Nod2^−/−^ mice correlates with the higher bacterial load observed in these mice at early time points after infection.

### Nod2-deficient mice elicit early enhanced production of inflammatory cytokines and chemokines during *A*. *baumannii* infection

**Figure 3:**
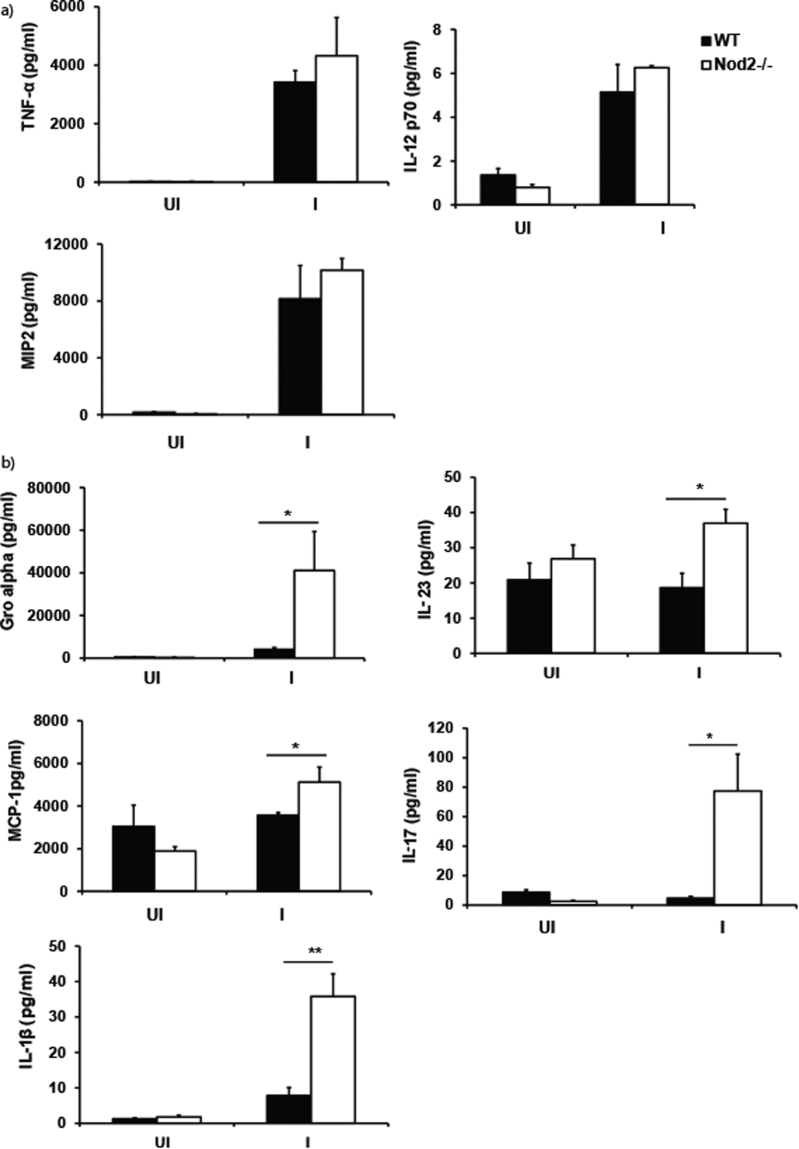
Nod2^−/−^ mice show heightened inflammatory cytokine response during *A*. *baumannii* infection. WT or Nod2^−/−^ C57BL/6 mice were intranasally instilled with *A*. *baumannii* (AB-19606) or PBS control (n = 4–5 mice per group). At 4 h post infection, mice were euthanized and BAL was obtained from these mice. Levels of various cytokines (**a**) TNF-α, IL-12p70, and MIP2 and (**b**) Gro-α, IL-23, MCP-1, IL-17 and IL-1β were analyzed in BAL by Luminex assay. *Represents *p* ≤ 0.05. Values shown represent mean ± standard deviation and are representative of two independent experiments with similar results. UI = uninfected PBS control; I = infected.

To gain insight into the mechanism of Nod2-mediated early *A*. *baumannii* protection, we next measured the lung inflammatory response to determine if the enhanced bacterial load seen in Nod2^−/−^ mice at 4 h post-infection is due to impaired production of cytokines/chemokines. There was no difference in the BAL levels of TNF-α, IL-12p70, and MIP2 in the infected Nod2^−/−^ mice in comparison to wild type controls (Fig. 3a). However, we found that the levels of Gro-α, IL-23, MCP-1, IL-17 and IL-1β were significantly increased in Nod2-deficient mice in comparison to wild type controls at 4 h post-infection (Fig. 3b). This result shows that the enhanced susceptibility of Nod2-deficient mice to *A*. *baumannii* pulmonary infection is not due to lack of cytokine/chemokine production at 4 h post-infection.

### Nod2-deficient mice generate reduced levels of ROS/RNS during early time points of *A*. *baumannii* infection

**Figure 4:**
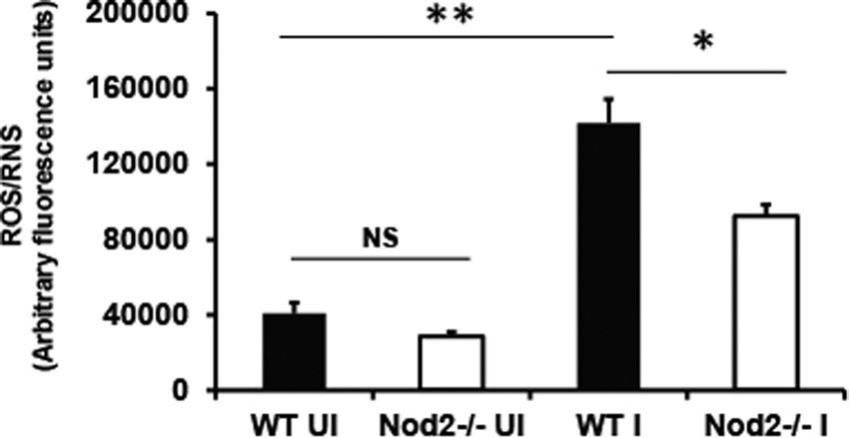
Lungs of *A*. *baumannii* infected Nod2^−/−^ mice show diminished ROS/RNS production as compared to lungs of infected WT mice. WT or Nod2^−/−^ mice were intranasally instilled with *A*. *baumannii* or PBS control (n = 4–5 mice per group). At 4 h post infection mice were euthanized. Lungs were assayed for presence of ROS and RNS as described in material and methods. *Represents *p* ≤ 0.05. **Represents *p* ≤ 0.01, NS denotes not significant. Values shown represent mean ± standard deviation of results of three independent experiments. UI = uninfected PBS control; I = infected.

Apart from cytokine production, other Nod2-mediated antibacterial responses include antimicrobial peptide production and ROS/RNS release. Therefore we next assessed if there is any defect in the production of anti-microbial factors previously known to restrict *A*. *baumannii* lung infection such as β-defensin 2 (BD2) and ROS/RNS in the absence of Nod2. Unfortunately, we were unable to detect any induction of BD2 in lungs of either wild type or Nod2-deficient mice with *A*. *baumannii* infection (not shown). However, we observed that the wild type mice elicited significant amounts of ROS/RNS in the lungs following *A*. *baumannii* infection at 4 h (*p* = 0.009) (Fig. 4). Moreover, Nod2^−/−^ mice showed significantly reduced production of ROS/RNS in comparison to wild type controls (Fig. 4). From this data, we conclude that at least one of the mechanisms by which Nod2 pathway contributes to the early *A*. *baumannii* control is through ROS/RNS production.

### Administration of Nod2 ligand muramyl dipeptide (MDP) protects the mice from *A*. *baumannii* pulmonary infection

**Figure 5:**
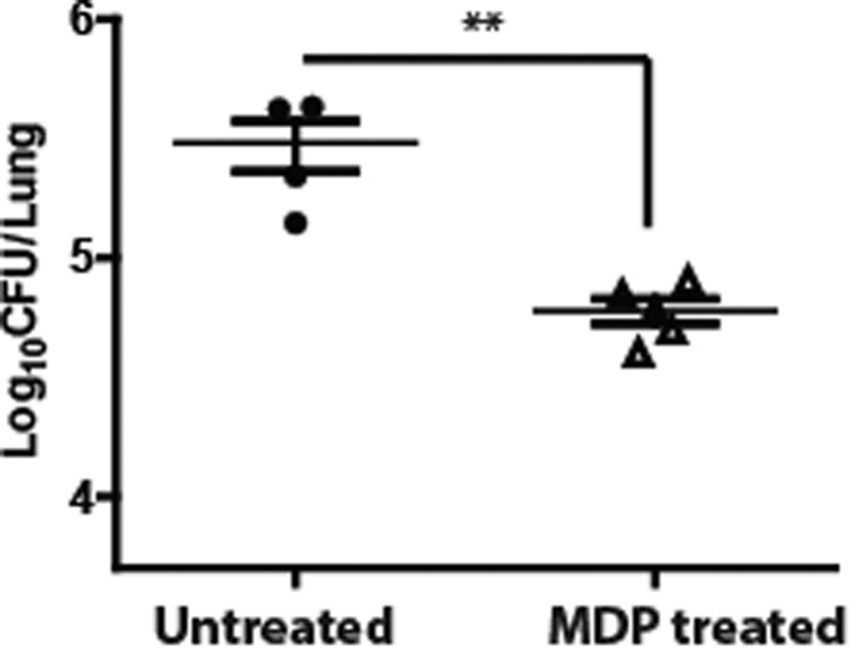
MDP administration protects mice from subsequent *A*. *baumannii* challenge. Wild type (WT) mice were administered PBS or MDP for 3 consecutive days as described in material and methods. At day 4, mice were infected with *A*. *baumannii* (n = 4–5 mice per group). The bacterial load was determined in lungs at 4 h post infection. The confirmed bacterial inoculation dose was 0.2 × 10^7^. **Represents *p* ≤ 0.01. Data are represented as mean ± standard deviation and are representative of three independent experiments with similar results.

Our published *in vitro* data showed that pre-stimulation of lung epithelial cells with ligands of Nod2 (muramyl dipeptide, MDP) protected the cells from subsequent *A*. *baumannii* infection^14^. The current study further demonstrates that Nod2 is involved in the early innate immune control of *A*. *baumannii in vivo*. Hence we reason that MDP priming could be used as a strategy to control *A*. *baumannii* infection in the lungs. To test this hypothesis, mice were administered intrperitoneally with either MDP or PBS (control) followed by *A*. *baumannii* infection. Remarkably, as seen in Fig. 5, we found that MDP-administered mice were significantly more resistant to subsequent *A*. *baumannii* pulmonary challenge (*p* = 0.007). Thus Nod2 priming could be considered as a strategy to control *A*. *baumannii* infection.

## Discussion

Based on our study, Nod2-deficient mice are more susceptible to *A*. *baumannii* at early, but not at late time points of infection. This indicates that Nod2 is involved in preventing the early colonization of *A*. *baumannii* in the lung. In addition, based on our previous *in vitro* study, a related receptor Nod1 is also involved in *A*. *baumannii* lung control; however, we didn’t examine the *in vivo* contribution of Nod1 pathway during *A*. *baumannii* infection in the current study. Because of the redundant roles of Nod1 and Nod2 signaling pathways, it is possible that a combined action of both Nod1 and Nod2 may be required for the effective control of *A*. *baumannii*. Future studies using Nod1/Nod2 double knockout mice or Rip2^−/−^ mice will clarify this point further.

Consistent with the higher bacterial load, *A*. *baumannii* infected Nod2^−/−^ mice also exhibited enhanced recruitment of neutrophils to the lungs in comparison to the wild type mice. MIP-2 and Gro-α (KC) are the two major chemokines involved in mediating the neutrophil recruitment to the lungs during infection. Although the levels of MIP-2 was comparable between wild type and Nod2^−/−^ mice (Fig. 3a), our results show that the levels of Gro-α (KC) was significantly higher in Nod2^−/−^ mice in comparison to wild type mice during *A*. *baumannii* infection (Fig. 3b). In addition, the levels of other known neutrophil chemoattractants such as IL-17 and IL-1β were also higher in Nod2^−/−^ mice in comparison to wild type mice during *A*. *baumannii* infection (Fig. 3b). We speculate that the enhanced production of these chemokines/cytokines in *A*. *baumannii* infected Nod2^−/−^ mice are responsible for the increased neutrophil recruitment observed in these mice. The mechanism by which Nod2 deficiency leads to the enhanced production of Gro-α, IL-17 and IL-1β during *A*. *baumannii* infection is currently unknown and will be a matter of future research.

Our results show that one of the mechanisms by which Nod2 pathway contributes to the early *A*. *baumannii* control is through the generation of microbicidal ROS/RNS. The relative contribution of ROS and RNS in *A*. *baumannii* immunity was examined in a previous study by another group^12^. Qiu *et al*. demonstrated that gp91phox^−/−^, but not NOS2^−/−^, mice were highly susceptible to intranasal *A*. *baumannii* infection implying that NADPH-dependent ROS production is essential for the effective early control of *A*. *baumannii* from the lungs, with RNS playing only a minimal or no role. Despite the importance of ROS in *A*. *baumannii* control, however, the pathways involved in the ROS generation during *A*. *baumannii* infection are not characterized yet. Based on our current study, we conclude that the ROS production during *A*. *baumannii* infection is mediated through Nod2 signaling pathway. Generation of the bactericidal ROS via Nod2-dependent pathway has been shown for other intracellular bacteria such as *Listeria monocytogenes* ^21^.

Although our studies showed that the ROS production was significantly reduced in Nod2^−/−^ mice in comparison to wild type controls following *A*. *baumannii* infection, the ROS production was not completely abrogated in Nod2^−/−^ mice, implying that additional innate immune pathways (such as Nod1 pathway) might also contribute to the ROS production during *A*. *baumnnii* lung infection. Although our previous *in vitro* study showed that Nod1-Nod2-Rip2 pathway contributes to intracellular *A*. *baumannii* control through the production of β-defensin 2, we were unable to detect any induction of β-defensin 2 in the lungs of either wild type or Nod2-deficient mice with *A*. *baumannii* infection, probably due to the induction of very low levels of detectable β-defensin 2 in our experimental system.

Although Nod2 is mainly expressed in lung epithelial cells, macrophages and neutrophils, the cell types involved in Nod2-mediated *A*. *baumannii*-induced ROS production is not examined in this study. While some earlier studies have demonstrated the importance of macrophages in the early innate control of *A*. *baumannii* ^8,22^, one study has reported that macrophages may play only a minor role in the innate host defense against *A*. *baumannii* ^9^. Hence we speculate that lung epithelial cells or neutrophils are the major potential cell types that may be involved in the Nod2-mediated ROS production during *A*. *baumannii* lung infection. However, since alveolar macrophages are the first line of resident immune cells that are capable of detecting and eliminating respiratory bacterial pathogens, their role in mediating the early induction of Nod2-mediated ROS during *A*. *baumannii* infection cannot be ruled out. A higher influx of neutrophils was observed in Nod2^−/−^ mice during *A*. *baumannii* infection at 4 h post-infection. Considering the importance of early neutrophil recruitment to the lungs in the control of *A*. *baumannii* infection, however, it is interesting to note that even this enhanced neutrophil numbers was unable to control the bacterial load at this time point. This implies that the microbicidal abilities of the recruited neutrophils may be compromised in the absence of Nod2.

Immunomodulatory therapies in patients with compromised immune status are promising strategies to prevent or treat *A*. *baumannii* infections. Nod1/Nod2 agonists have been shown to have both immunomodulatory and anti-infective properties. The Nod2 ligand MDP alone or combinations with other agents have long been recognized as immunostimulants to induce non-specific immune response to antigens or against various pathogens including bacteria^23–26^. Since MDP is toxic to be used as adjuvant in humans, a safe synthetic alternative of MDP called murabutide was developed and it has been used extensively in preclinical animal and human clinical trials previously^24,27–31^. Since our studies identified that administration of the Nod2 ligand MDP confers protection to *A*. *baumannii* pulmonary challenge, it will be important to further explore the possibility of manipulating Nod2 pathway as an immunomodulatory strategy to prevent or treat *A*. *baumannii* infections.

In conclusion, our study has revealed a novel mechanism by which early *A*. *baumannii* load is kept in check by the lung innate immune system. NOD agonists could be considered as immunostimulants or anti-infectives against *A*. *baumannii* pneumonia.

## Materials and Methods

### Ethics Statement

All animal experiments were performed in strict accordance with the prevailing Singapore National Advisory Committee for Laboratory Animal Research (NACLAR) guidelines and approved by Sing-Health Institutional Animal Care and Use Committee, Singapore (Protocol #: 2013/SHS/863).

### Mice

Eight-twelve weeks old female mice were used for all the mice infection experiments. C57BL/6 J wild type mice were purchased from the Biological Resource Center ((BRC), Agency for Science, Technology, and Research (A*STAR), Singapore). Nod2^−/−^ mice were obtained from The Jackson Laboratory (B6.129S1-Nod2^tm1Flv^/J, Stock #005763) and were backcrossed to C57BL/6 J background for at least 10 generations.

### Reagents

Tryptic soy broth (22092) and tryptic soy agar (22091) were purchased from Sigma-Aldrich. MDP (tlrl-mdp) was obtained from InvivoGen. MPO antibody (Ab-9535) was purchased from Abcam and was used at 1:25 dilution for immunohistochemistry. OxiSelect™ *In Vitro* ROS/RNS Assay Kit (STA-347) was obtained from Cell Biolabs, Inc. (San Diego, USA). Beta defensin 2 ELISA kit (MBS2602457) was purchased from MyBiosource. All reagents were used according to manufacturer’s instructions.

### Bacterial strains and growth conditions

*A*. *baumannii* ATCC 19606 strain was grown overnight in Tryptic soy broth supplemented with 25 µg/ml streptomycin at 37 °C with shaking at 220 rpm.

### A. baumannii *in vivo* infection

Mice were infected with *A*. *baumannii* (AB-19606) intranasally as described before^18^. Briefly, bacteria were grown overnight in tryptic soy broth supplemented with 25 μg/ml streptomycin and the cultures were re-inoculated in tryptic soy broth at 1%. Three hours later bacteria were harvested by centrifugation (3,000 × g, 10 min), washed twice with PBS, and the bacteria were resuspended at indicated CFUs in 40 μl PBS. Actual inoculum concentrations were determined by plating serial dilutions on TSA plates supplemented with 25 μg/ml streptomycin. Mice were anaesthetized by administering ketamine/xylazine intraperitoneally and were subsequently infected intranasally with *A*. *baumannii*.

### *In vivo* bacterial load enumeration

For enumeration of bacterial CFU in lungs, mice were sacrificed at indicated time points post infections. Lungs were removed aseptically and were homogenized in 0.1% triton X-100. Serial dilutions of the lysates were plated on TSA streptomycin plates for CFU enumeration.

### Collection of broncheoalveolar lavage and cytokine analysis

For the aspiration of broncheoalveolar lavage (BAL), trachea was exposed through a midline incision. Lungs were lavaged 4–5 times with 1 ml PBS injected through the trachea. BAL fluid was spun at 2000 rpm for 10 minutes. Supernatants were concentrated using Vivaspin 500 columns (5 kDa cutoff, VS0012, Sartorius) and were analyzed for various cytokines using ProcartaPlex® Multiplex Immunoassay from eBioscience by the Immunomonitoring Platform, SIgN, A*STAR, Singapore.

### Immunohistochemistry

Lungs were insufflated with 10% neutral buffer formalin via trachea and removed en bloc. The lungs were kept in 10% neutral buffer formalin until further processing for histological studies which was carried out at histopathology laboratory at The Advanced Molecular Pathology Laboratory (AMPL), Institute of Molecular and Cell Biology (IMCB), Singapore. Lungs were embedded in paraffin blocks. Serial sections (4 to 5 µm) were cut and processed for H&E staining or MPO staining by immunohistochemistry (IHC).

H&E slides were analyzed under light microscopy by a pathologist in a blind fold analysis. Sections were evaluated for the following parameters: (a) alveolar/peribronchial neutrophilic infiltrates, (b) vasculitis and vascular extravasation and (c) bronchial epithelial sloughing/necrosis. Sections were scored as following for neutrophil influx: (1) Minimal influx (2) Mild influx (3) Moderate influx.

IHC slides were scanned using Leica SCN400 slide scanner (Leica Microsystems, Germany). Images were exported to Slidepath Digital Image Hub (Leica Microsystems, Germany) for viewing. Images were analyzed using Slidepath Tissue IA 2.0 software (Leica Microsystems, Germany). Data was collated using Microsoft Excel.

### MDP treatment of mice

Mice were administered intraperitoneally with either PBS or MDP (100 μg) for 3 consecutive days as described before^32^. At day 4, mice were infected with 2 × 10^7^ *A*. *baumannii* as described above. Mice were sacrificed at 4 hours post infection and bacterial load in lungs was determined as described above.

### ROS/RNS assay

Lungs were homogenized in PBS with protease inhibitor. The lysates were then spun at 2000 rpm for 10 minutes. The supernatant was assayed for ROS/RNS using OxiSelect™ *In Vitro* ROS/RNS Assay Kit.

### Statistical analysis

P values were determined by unpaired two-tailed Student’s t test. P values less than 0.05 were considered statistically significant

## References

[1] Antunes LC, Visca P, Towner KJ (2014). Acinetobacter baumannii: evolution of a global pathogen. Pathog Dis.

[2] Clark NM, Zhanel GG, Lynch JP (2016). Emergence of antimicrobial resistance among Acinetobacter species: a global threat. Curr Opin Crit Care.

[3] Fournier PE, Richet H (2006). The epidemiology and control of Acinetobacter baumannii in health care facilities. Clinical infectious diseases: an official publication of the Infectious Diseases Society of America.

[4] Falagas ME, Karveli EA, Kelesidis I, Kelesidis T (2007). Community-acquired Acinetobacter infections. Eur J Clin Microbiol Infect Dis.

[5] Joly-Guillou ML (2005). Clinical impact and pathogenicity of Acinetobacter. Clin Microbiol Infect.

[6] Breslow JM (2011). Innate immune responses to systemic Acinetobacter baumannii infection in mice: neutrophils, but not interleukin-17, mediate host resistance. Infection and immunity.

[7] van Faassen H (2007). Neutrophils play an important role in host resistance to respiratory infection with Acinetobacter baumannii in mice. Infection and immunity.

[8] Qiu H (2012). Role of macrophages in early host resistance to respiratory Acinetobacter baumannii infection. PloS one.

[9] Tsuchiya T (2012). NK1.1(+) cells regulate neutrophil migration in mice with Acinetobacter baumannii pneumonia. Microbiol Immunol.

[10] Knapp S (2006). Differential roles of CD14 and toll-like receptors 4 and 2 in murine Acinetobacter pneumonia. American journal of respiratory and critical care medicine.

[11] March C (2010). Dissection of host cell signal transduction during Acinetobacter baumannii-triggered inflammatory response. PloS one.

[12] Qiu H, Kuolee R, Harris G, Chen W (2009). Role of NADPH phagocyte oxidase in host defense against acute respiratory Acinetobacter baumannii infection in mice. Infection and immunity.

[13] Kim CH (2014). Tolllike receptor 2 promotes bacterial clearance during the initial stage of pulmonary infection with Acinetobacter baumannii. Molecular medicine reports.

[14] Bist P (2014). The Nod1, Nod2, and Rip2 axis contributes to host immune defense against intracellular Acinetobacter baumannii infection. Infection and immunity.

[15] Choi CH, Lee JS, Lee YC, Park TI, Lee JC (2008). Acinetobacter baumannii invades epithelial cells and outer membrane protein A mediates interactions with epithelial cells. BMC microbiology.

[16] Smani Y, Docobo-Perez F, McConnell MJ, Pachon J (2011). Acinetobacter baumannii-induced lung cell death: role of inflammation, oxidative stress and cytosolic calcium. Microb Pathog.

[17] Noto MJ (2015). Toll-Like Receptor 9 Contributes to Defense against Acinetobacter baumannii Infection. Infection and immunity.

[18] Dikshit N (2017). NLRP3 inflammasome pathway has a critical role in the host immunity against clinically relevant Acinetobacter baumannii pulmonary infection. Mucosal Immunol.

[19] Frutuoso MS (2010). The pattern recognition receptors Nod1 and Nod2 account for neutrophil recruitment to the lungs of mice infected with Legionella pneumophila. Microbes and infection/Institut Pasteur.

[20] Shimada K (2009). The NOD/RIP2 pathway is essential for host defenses against Chlamydophila pneumoniae lung infection. PLoS pathogens.

[21] Lipinski S (2009). DUOX2-derived reactive oxygen species are effectors of NOD2-mediated antibacterial responses. J Cell Sci.

[22] Bruhn KW (2015). Host fate is rapidly determined by innate effector-microbial interactions during Acinetobacter baumannii bacteremia. J Infect Dis.

[23] Bertot GM, Becker PD, Guzman CA, Grinstein S (2004). Intranasal vaccination with recombinant P6 protein and adamantylamide dipeptide as mucosal adjuvant confers efficient protection against otitis media and lung infection by nontypeable Haemophilus influenzae. J Infect Dis.

[24] Geddes K, Magalhaes JG, Girardin SE (2009). Unleashing the therapeutic potential of NOD-like receptors. Nature reviews. Drug discovery.

[25] Hancock RE, Nijnik A, Philpott DJ (2012). Modulating immunity as a therapy for bacterial infections. Nature reviews. Microbiology.

[26] Tatara O, Nakahama C, Niki Y (1992). Synergistic effects of romurtide and cefmenoxime against experimental Klebsiella pneumonia in mice. Antimicrobial agents and chemotherapy.

[27] Bahr GM (2003). Non-specific immunotherapy of HIV-1 infection: potential use of the synthetic immunodulator murabutide. J Antimicrob Chemother.

[28] Darcissac EC (2000). The synthetic immunomodulator murabutide controls human immunodeficiency virus type 1 replication at multiple levels in macrophages and dendritic cells. Journal of virology.

[29] De La Tribonniere X (2003). A phase I study of a six-week cycle of immunotherapy with Murabutide in HIV-1 patients naive to antiretrovirals. Med Sci Monit.

[30] Telzak E (1986). Clinical evaluation of the immunoadjuvant murabutide, a derivative of MDP, administered with a tetanus toxoid vaccine. J Infect Dis.

[31] Vidal, V. F. *et al*. Macrophage stimulation with Murabutide, an HIV-suppressive muramyl peptide derivative, selectively activates extracellular signal-regulated kinases 1 and 2, C/EBPbeta and STAT1: role of CD14 and Toll-like receptors 2 and 4. *Eur J Immunol* **31**, 1962–1971, https://doi.org/10.1002/1521-4141(200107)31:7<1962::AID-IMMU1962>3.0.CO;2-V (2001). 10.1002/1521-4141(200107)31:7<1962::aid-immu1962>3.0.co;2-v 11449348

[32] Watanabe T (2008). Muramyl dipeptide activation of nucleotide-binding oligomerization domain 2 protects mice from experimental colitis. J Clin Invest.

